# Microbiome, Mycobiome and Related Metabolites Alterations in Patients with Metabolic Syndrome—A Pilot Study

**DOI:** 10.3390/metabo12030218

**Published:** 2022-02-28

**Authors:** Gratiela Gradisteanu Pircalabioru, Iuliana Ilie, Luciana Oprea, Ariana Picu, Laura Madalina Petcu, Liliana Burlibasa, Mariana-Carmen Chifiriuc, Madalina Musat

**Affiliations:** 1 Research Institute of University of Bucharest (ICUB), 050095 Bucharest, Romania; gratiela.gradisteanu@icub.unibuc.ro; 2 Faculty of Biology, University of Bucharest, 050095 Bucharest, Romania; liliana.burlibasa@bio.unibuc.ro; 3 Gral Medical Clinic, 031424 Bucharest, Romania; ilieiu@yahoo.com; 4 National Institute of Endocrinology C.I. Parhon, 011863 Bucharest, Romania; lucianaoprea92@yahoo.com (L.O.); mdmusat@yahoo.com (M.M.); 5 “N.C. Paulescu” National Institute of Diabetes, Nutrition and Metabolic Diseases, 020042 Bucharest, Romania; 6 Academy of Romanian Scientists, 010071 Bucharest, Romania; 7 Department of Endocrinology, Carol Davila University of Medicine and Pharmacy, 020021 Bucharest, Romania

**Keywords:** metabolic syndrome, microbiome, microbiota, mycobiome, metabolome

## Abstract

Metabolic syndrome (MetSyn) has a rapidly growing worldwide prevalence, affecting over 1 billion people. MetSyn is clustering many pathological conditions, which, untreated, could increase the risk and often lead to more severe metabolic defects such as type 2 diabetes and non-alcoholic fatty liver disease. Many data demonstrate the complex role of gut microbiota in the host metabolism, and hence, deciphering the microbiome patterns linked to MetSyn could enable us for novel diagnosis and monitoring markers and for better disease management. Moreover, interventions designed to alter patient microbiome composition may help prevent or decrease morbidity linked with MetSyn. However, the microbiome composition is largely different across geographically distinct populations. Our study investigated the microbiota and mycobiome patterns in Romanian metabolic syndrome patients. We also correlated the identified microbiome–mycobiome patterns with levels of metabolites important for host health such as short chain fatty acids, organic acids, and taurine. We found that MetSyn patients are harboring a microbiome enriched in *Enterobacteriaceae*, *Turicibacter* sp., *Clostridium coccoides,* and *Clostridium leptum,* while beneficial taxa such as *Butyricicoccus* sp., *Akkermansia muciniphila*, and *Faecalibacterium prausnitzii* were decreased. These microbiome changes were correlated with lower butyrate levels and increased succinate. In terms of mycobiome signatures, MetSyn was associated with a high abundance of *Saccharomyces* and *Aspergillus* species. Our data are the first reported on a Romanian population and confirming that the pathogenesis of MetSyn is closely related to gut microbiome and homeostasis.

## 1. Introduction

Metabolic syndrome (MetSyn) is a combination of interconnected biochemical, physiological, clinical, and metabolic factors characterized by high blood pressure, raised fasting glucose, dyslipidemia, and central obesity [1]. While specific diagnostic criteria may differ, in all cases MetSyn is undoubtedly associated with increased risk of mortality and comorbidities (i.e., cardiovascular disease and type 2 diabetes). MetSyn prevalence is rapidly growing worldwide, with some estimates suggesting that over 1 billion people are affected by this syndrome [1] and with children and adolescents being increasingly affected nowadays [2]. According to the PREDATORR study, in a cohort of 2681 Romanian subjects aged 20–79 years the prevalence of MetSyn was 38.50% [3].

These rising rates of MetSyn are to a great extent attributed to high-calorie diets and sedentary lifestyles. Nevertheless, the precise physiological mechanisms driving MetSyn development are largely unknown. As revealed by both animal and human studies, a pathogenic trigger affecting host metabolic balance is represented by the gut microbiota. The microbiota orchestrates several aspects crucial for host metabolic functions, including modulation of nutrition and energy harvest, gut motility, glucose and lipid metabolism, appetite, energy absorption, and hepatic fatty storage [4,5].

Disturbances in the host–microbiome communication trigger the intestinal translocation of microbial fragments and the development of “metabolic endotoxemia”, culminating in systemic inflammation and insulin resistance [6]. Moreover, diets rich in in processed foods were shown to alter microbiome composition in ways that promote insulin resistance and higher fat mass, probably due to enhanced energy production yield after digestion [7]. Microbiome diversity is decreased as a result of a sedentary lifestyle, a fact which triggers elevated inflammation and metabolic disease risk [8].

In this context, interventions designed to alter patient microbiome composition (probiotics, prebiotics, postbiotics, symbiotics, faecal microbiota transplantation, exercise, etc.) may help prevent or decrease morbidity linked with MetSyn [7,9,10,11]. However, many studies have shown that the composition of the human gut microbiome is largely different across geographically distinct populations, probably related to many factors, such as diet, lifestyle, socio-economic status, etc. These differences could also be translated into differences in susceptibility to different diseases, including MetSyn [12]. Thus, deciphering the microbiome patterns linked to MetSyn in different populations could enable us for more targeted gut microbiota-targeted interventions, contributing to a better management of MetSyn patients in different countries.

The gut mycobiome is made up from the fungi residing in the intestinal tract, and it accounts for ~0.1% of the gut microbiota [13]. Typically, the human gut mycobiome is dominated by *Saccharomyces, Malassezia*, and *Candida*. Studies in mice with dextran sodium sulphate (DSS)-induced colitis suggest a role for *Candida albicans* or *Saccharomyces cerevisiae* in maintaining gut homeostasis [14]. A comprehensive characterization of the gut commensal mycobiome is lacking in people with various ailments.

Our pilot study investigated for the first time the microbiota and mycobiome patterns in Romanian patients with MetSyn. Fecal samples were collected from MetSyn patients (*n* = 30) and healthy controls (*n* = 30) and further used for bacterial DNA isolation. Using 16 rDNA qRT-PCR, we analyzed phyla abundance as well as the relative abundance of specific bacterial (*Lactobacillus* sp., *Enterobacteriaceae*, *Ruminococus* sp., *Faecalibacterium* sp., *Clostridium coccoides,* and *Clostridium leptum*) and fungal groups (*Candida* sp., *Aspergillus* sp., and *Saccharomyces* sp.). We also correlated the identified microbiome-mycobiome patterns with the levels of metabolites important for host health such as short chain fatty acids (SCFAs), organic acids (lactate), and taurine.

## 2. Results

Our study aimed to investigate the microbiota and mycobiome patterns in Romanian MetSyn patients and to correlate the identified microbial signatures with levels of metabolites (short chain fatty acids, organic acids, and taurine) known to be dependent on gut microbiota eubiosis and important for maintaining human host homeostasis and health condition. Our pilot study enrolled 30 MetSyn patients and 30 healthy controls. Among the 30 MetSyn patients investigated, 28 had impaired glucose tolerance, out of which 22 had type 2 diabetes. The patients characteristics including age, BMI, lipid profiles, as well as treatment regimens are all listed in Table 1. There were no statistically significant differences in the gender composition of the two groups. The parameters BMI, HbAc, TG, HDL, and LDL were significantly (*p* < 0.05) higher in the MetS group compared with the control group.

We compared the fecal microbiota of MetSyn patients with that of healthy volunteers using qPCR of the 16S rRNA gene. The qPCR analysis was done using SYBR Green primers recognizing different phyla but also bacterial families. Primers for the Universal Eubacteria 16S were used for normalization. We also investigated the metabolome patterns as well as the abundance of different fungi (the mycobiome) in the stool samples.

**Figure 1:**
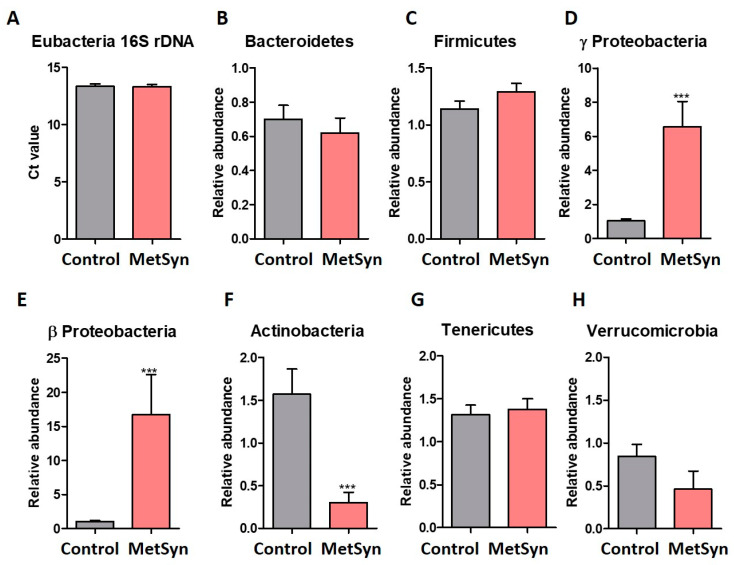
Microbial phyla analysis in MetSyn patients (*n* = 30) versus healthy controls (*n* = 30). (**A**) Total bacteria represented as Ct values obtained from qRT-PCR targeting the 16S rDNA of all Eubacteria. The abundance of the Bacteroidetes (**B**), Firmicutes (**C**), Gamma Proteobacteria (**D**), Beta Proteobacteria (**E**), Actinobacteria (**F**), Tenericutes (**G**), and Verrucomicrobia (**H**) phyla in fecal samples harvested from healthy individuals and MetSyn patients; *** *p* < 0.0001, Mann–Whitney test.

The total amount of bacteria in stool samples are represented as Real Time PCR threshold cycle values (Ct) in Figure 1A. Both healthy controls and MetSyn patients had similar levels of Eubacteria (Figure 1A). The gut microbiome is dominated by members of the Gram-positive *Firmicutes* and the Gram-negative *Bacteroidetes* phyla, followed by several others phyla, including *Proteobacteria, *Actinobacteria,** and *Verrucomicrobia* [15]. Although many data from animal models and human studies reported differences in the two dominant bacterial phyla with a significant increase in the *Firmicutes* and decrease in the *Bacteroidetes* levels in obesity [16], we did not observe significant differences between the two phyla (Figure 1B,C), even though MetSyn patients exhibited a tendency to harbor less *Bacteroidetes* and more *Firmicutes*. In our study, no significant differences were recorded in the case of the Firmicutes-to-Bacteroidetes ratio (Supplementary Figure S1).

Notably, stool samples collected from MetSyn patients were enriched in *Proteobacteria* families, particularly in *Gamma Proteobacteria* (Figure 1D) and *Beta Proteobacteria* (Figure 1E). *Actinobacteria* are one the four major phyla of the intestinal microbiota, and despite the fact they represent only a small percentage, they play a pivotal role in maintaining gut homeostasis [17]. The MetSyn patients enrolled in our study were significantly depleted in *Actinobacteria* (Figure 1F), suggesting the loss of beneficial taxa (e.g., bifidobacteria).

Investigation of other microbiota phyla such as *Tenericutes* and *Verrucomicrobia* showed no statistical significance between the two groups (Figure 1G,H). Next, we analyzed the abundance of different bacterial populations of the gut microbiome.

**Figure 2:**
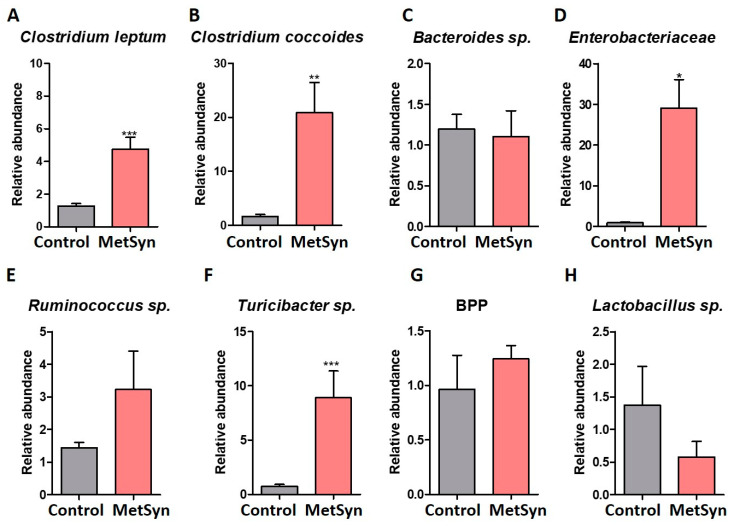
Bacterial population analysis in MetSyn patients (*n* = 30) versus healthy controls (*n* = 30). The relative abundance of *Clostridium leptum* (**A**), *Clostridium coccoides* (**B**), *Bacteroides* sp. (**C**), *Enterobacteriaceae* (**D**), *Ruminococcus* (**E**), *Turicibacter* (**F**), *Bacteroides-Porphyromonas-Prevotella* (**G**), and *Lactobacillus* sp. (**H**) in fecal samples harvested from healthy individuals and MetSyn patients; * *p* < 0.05, ** *p* < 0.005, *** *p* < 0.0001, Mann–Whitney test.

The levels of *Clostridium leptum* and *Clostridium coccoides* group were significantly higher in MetSyn patients (Figure 2A,B), whereas no differences were observed in case of *Bacteroides* species abundance (Figure 2C). The strictly anaerobic *Clostridium coccoides* group represents 25% to 60% of the total microbiota. This group is comprised of genera such as *Clostridium*, *Blautia*, *Roseburia, Anaerostipes*, *Ruminococcus*, *Dorea*, and *Eubacterium* [18].

Specific taxonomic shifts have been linked to intestinal inflammation, including a relative increase in the abundance of *Enterobacteriaceae* [19]. Importantly, the microbiota of Romanian patients with MetSyn was significantly enriched in *Enterobacteriaceae* (Figure 2D) in accordance with the increased abundance of *Gamma Proteobacteria* (Figure 1D).

MetSyn patients showed higher abundance of *Ruminococcus* sp., but this difference was not statistically significant between the two study groups (Figure 1E). Moreover, the gut microbiome of MetSyn subjects was significantly enriched in *Turicibacter* sp. (Figure 1F), a member of the microbiota associated with the inflammatory status [20,21,22] that could act as an opportunistic pathogen [23].

The three predominant *Bacteroidetes* genera of the human gastrointestinal tract are represented by *Bacteroides*, *Prevotella*, and *Porphyromonas* (BPP). In our analysis, no significant differences were observed in faecal BPP levels between MetSyn patients and healthy controls (Figure 2G).

Considering the well-known benefits of lactobacilli for host health [24,25] we also investigated whether MetSyn patients were depleted from his beneficial taxa. Indeed, MetSyn patients harbored less lactobacilli, but this difference was not statistically significant (Figure 2H).

**Figure 3:**
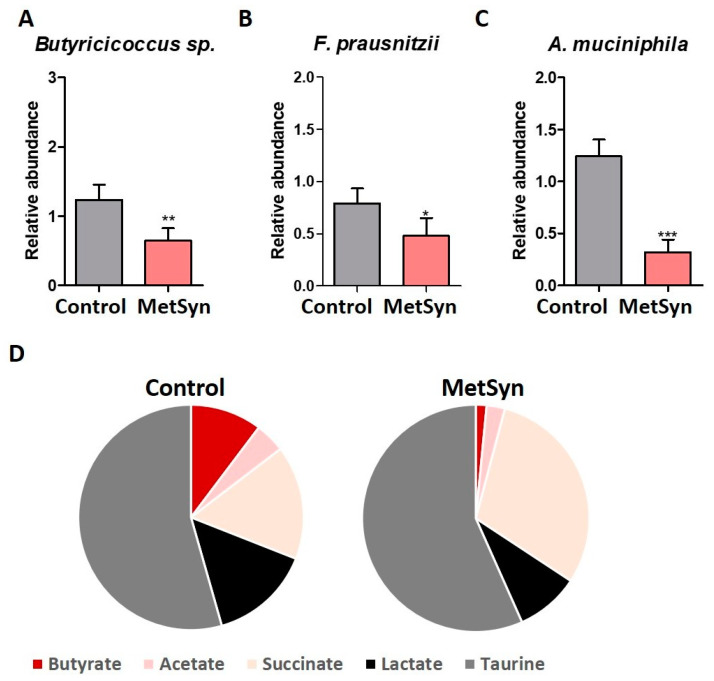
Microbiome-metabolome features in Met Syn. Relative abundance of *Butyricicoccus* sp. (**A**), *F. prausnitzii* (**B**), and *A. muciniphila* (**C**) in Met Syn patients; (**D**) Metabolite analysis in Healthy controls (*n* = 30) and MetSyn patients (*n* = 30); * *p* < 0.05, ** *p* < 0.005, *** *p* < 0.0001, Mann–Whitney test.

In our study the microbiome of MetSyn patients was also characterized by significantly lower levels of *Butyricicoccus* sp., a bacterial population known to be an important producer of butyrate, a SCFA involved in host intestinal homeostasis (Figure 3A). Moreover, MetSyn patients were low in *Faecalibacterium prausnitzii* (Figure 3B), another microbe that produces butyrate and that is known for its association with gut health [26]. These results were correlated with those obtained for the metabolite levels in the stool samples collected from Metsyn patients and healthy controls (Figure 3D). MetSyn patients harbored significantly lower levels of butyrate when compared to the healthy controls (Figure 3D) (*p* value 0.0018, Mann–Whitney test).

MetSyn patients’ feces were enriched in succinate (*p* < 0.0001) and taurine (*p* = 0.0003), whereas acetate and lactate levels were similar compared to the healthy individuals (Figure 3D). *Akkermansia muciniphila* is a mucin-degrading bacterium colonizing the human gut. *A. muciniphila* is a potential probiotic that has been shown to exhibit protective effects against metabolic disorder, obesity, diabetes, and inflammation [27]. *A. muciniphila* abundance was consistently lower in all analyzed MetSyn subjects (Figure 3C).

**Figure 4:**
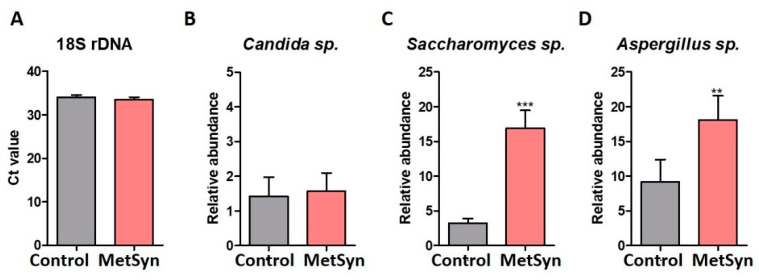
Fungal microbiome in MetSyn: total abundance of fungal 18SrDNA (expressed as Ct values) (**A**). *Candida* sp. (**B**), *Saccharomyces* sp. (**C**), *Aspergillus* sp. (**D**) abundance in fecal samples harvested from healthy individuals and MetSyn patients; *Saccharomyces* sp.; ** *p* < 0.01 *** *p* < 0.0001, Mann–Whitney test.

For the first time, this study also investigated the differences in the fungal microbiome of MetSyn patients. The total amount of fungal DNA was quantified in the fecal samples using universal primers for fungal 18S rDNA and the relative abundance of fungal populations such as *Candida* sp. and *Saccharomyces* sp. using specific primers (Figure 4A–D). Although we did not observe any statistically significant differences regarding the total amount of fungal DNA sequences or *Candida* sp. levels (Figure 4A,B), significantly higher levels of *Saccharomyces* sp. (Figure 4C) and *Aspergillus* sp. (Figure 4D) were found in MetSyn patients.

## 3. Discussion

Understanding the characteristics of the “healthy microbiome” is a major challenge in microbiota research. We are in a continuous process of understanding how the microbiome varies among apparently healthy people, how it is impacted by age, sex, ethnicity, geography, lifestyle, diet, and medication. Adding a new level of complexity, many microorganisms, including phages, viruses, fungi, and archaea, exist in the gut, but their toll to health and disease is largely unknown. Thus, defining the healthy microbiome is a dynamic and complex task. This investigation follows the line of many studies reported in the literature, trying to compare microbiome patterns between apparently healthy individuals to that of individuals with clearly documented ailments. Our pilot study performed on 30 individuals with MetSyn and 30 healthy controls highlights some distinct features in the gut microbiome related with this emerging pathology.

Large metagenome-wide studies have described microbiota imbalance (dysbiosis) in patients with obesity and type 2 diabetes. Even though microbiota composition changes identified in various studies and geographically distinct populations were different, some common findings were an increase in opportunistic pathogens and a reduction in butyrate-producing bacteria [28,29]. We show that the Romanian MetSyn patients harbor increased *Enterobacteriaceae* in their gut microbiome, similar to gut chronic inflammatory conditions, such as IBD [19]. Additionally, we previously reported that type 2 diabetes patients have a microbiome enriched in *Enterobacteriaceae* [20]. These facultative aerobic microorganisms are considered a marker of dysbiosis and gut inflammation [19]. Intestinal inflammation is characterized by increased blood flow and vascular permeability, which altogether lead to increased translocation of gut microbes, but also of other molecules, such as microbial lipopolysaccharides, trimethylamine, and other metabolites in the internal environment, contributing to the chronic inflammation associated with different conditions seen in patients with MetSyn, such as fat liver, macrophage infiltration in adipose tissue, cardiovascular disease development, insulin resistance, etc. The inflammatory response is more probably occurring in case of a Gram-negative enriched intestinal microbiota. The enrichment in *Enterobacteriaceae* leads to increased oxygen levels in the intestinal lumen as well [30]. This shift in oxygen levels subsequently halts the growth of obligate anaerobes (i.e., *Clostridium* groups IV or XIVa) and favors expansion of oxygen-tolerant species including aerobes and facultative anaerobes (*Enterobacteriaceae*), which maintain this vicious cycle of chronic inflammation and subsequent metabolic changes [31,32].

Short chain fatty acids (SCFAs) such as butyrate, propionate, and acetate produced primarily from the microbial fermentation of dietary fiber are thought to be key mediators of the beneficial effects of the gut microbiome [33,34]. SCFA directly modulate host health through tissue-specific mechanisms related to glucose homeostasis, gut barrier function, immunomodulation, and appetite regulation [35]. A detailed understanding of SCFA metabolism by the gut microbiome is pivotal to implement effective therapeutic strategies for microbiota modulation in diseased individuals.

Among SCFAs produced by the gut microbiota, it has been postulated that butyrate and propionate may improve glycaemia [36]. Butyrate and propionate elevate intestinal gluconeogenesis. Mouse studies show that increased intestinal gluconeogenesis promotes a reduction in hepatic gluconeogenesis, appetite, and weight, culminating in improved glucose homeostasis [37,38]. Similar to previous studies [28,36,39], we show that MetSyn patients are characterized by a microbiota low in butyrate producers.

Although propionate is less frequently studied compared to other microbial metabolites (i.e., butyrate), it harbors some distinct health-promoting properties including cholesterol-lowering, antiproliferative, and antilipogenic effects [40,41]. We observed no statistically significant difference regarding the abundance of propionate producing microbes such as *Prevotella* and Bacteroides [42]. For our subsequent studies, we aim to analyze propionate levels using nuclear magnetic resonance (NMR) or gas chromatography. Lactic acid is an organic compound produced mainly by lactobacilli [43]. Quantification of this organic acid (lactate) by spectrophotometric analysis revealed no significant difference between the two analyzed study cohorts. This is in accordance with the fact that both groups were similar in terms of lactobacilli abundance in the gut microbiota.

Succinate is a metabolite produced by both the host and the microbiota that can initiate important protective mechanisms in response to metabolic stress or tissue damage. Paradoxically, succinate can also accumulate under conditions of inflammation and microbiota disruption in the intestine, potentially promoting the expansion of potentially pathogenic microbes that exploit this metabolite as a nutrient source [44]. In our study, we found that MetSyn patients had higher levels of succinate, which may indicate a possible link with dysbiosis.

Our study identified a high abundance of clostridia (*C. leptum* and *C. coccoides* group) in the gut of MetSyn patients, a taxonomical trait that was associated with an excess of faecal bile acids [45]. In accordance, we found that the same patients had increased taurine levels, an aminoacid paramount for conjugation of bile acids. Interestingly, taurine was reported to play a very important role in energy metabolism and possibly a role in metabolic syndrome [46]. Nevertheless, we quantified fecal and not serum taurine. High levels of taurine were reported to be protective against coronary heart disease among individuals with high serum cholesterol levels [47]. It would definitely be interesting to quantify this metabolite also in the serum in a subsequent study with a larger patient cohort.

The gut mycobiome represents only a small fraction of the host microbiome, but it may exert an important impact on host health. Even though fungi make up a very small portion of the microbiota, they harbor decisive roles in gut homeostasis and the mucosal immune responses. Moreover, an interkingdom communication between bacteria and fungi has been suggested [48]. For instance, the presence of *Salmonella enterica serovar Typhimurium* has been shown to reduce the viability of and colonization with *Candida albicans*. In inflammatory bowel disease (IBD) patients, an interaction between gut bacteria and fungi has been hypothesized [49]. The mycobiome may trigger mucosal inflammation, providing a niche for overgrowth of pathobionts in IBD patients [48,50].

We found that MetSyn is linked with a high abundance of the commensal fungal taxa *Saccharomyces* and *Aspergillus*. *Aspergillus* is a genus consisting of several mold species and is a member of respiratory and gut mycobiome. *Aspergillus species* produce aflatoxins and may trigger opportunistic infections in humans. In the gut, high abundance of *Aspergillus* was correlated with an accentuated inflammatory response and increased colitis severity [51].

The low abundance of *A. muciniphilia* in all the analyzed MetSyn subjects suggests a common pathway of possible intervention in reducing body weight and insulin resistance through colonization with *Akkermansia* spp., as numerous studies in the recent years observed benefits of *A. muciniphilia* supplementation strategies [52]. Other approachs such as AI-prediction of gut microbiome response to dietary composition has shown promising results in segregating favorable taxa for cardiometabolic health [53].

The main limitation of this study is the low number of the analyzed patients, which have started to be enrolled just before the onset of COVID-19 pandemic. As we know from the literature as well as from our own ongoing research, COVID-19 triggers some important changes in the microbiome; therefore, we chose to discontinue the recruitment. Moreover, we used stringent exclusion criteria for patients selection. Despite this limitation, the obtained results were statistically significant and demonstrate that MetSyn patients harbor gut microbiome alterations correlated with changes in the profile of related metabolites.

In this pilot study, we analyzed certain subsets of microorganisms and some of the metabolites they produce. Nevertheless, we employed the use of targeted identification of certain bacterial and fungal taxa instead of 16S/18S rRNA sequencing, so there is the possibility of other differences that may exist between the microbes correlated with MetSyn. Our study is the first one showing mycobiome alterations in MetSyn patients. Since the host–fungi–bacteria interplay is largely unknown, investigation of the fungal signatures in various ailments may provide valuable insights into the role of the mycobiome in the pathophysiology of these diseases, potentially enabling improved treatment strategies.

## 4. Materials and Methods

### 4.1. Study Group

The study population (*n* = 60) was represented by 30 patients diagnosed with MetSyn from the National Institute of Endocrinology “C. I. Parhon” (16 patients) and “N.C. Paulescu” National Institute of Diabetes, Nutrition, and Metabolic Diseases (14 patients), Bucharest, Romania and 30 healthy volunteers. The two hospitals involved in the study are reference centers for treatment of patients with MetSyn and diabetes in Romania, the patients admitted coming from various parts of the country. All participants received and signed an informed consent, and the Ethical Committee approved the study (CEC reg. NO. 235/9.10.2019).

Inclusion criteria for participating in the study were: (1) diagnosis of MetSyn using the International Federation Of Diabetes criteria, 2006 [54]; waist >  94 cm (men) or >80 cm (women) along with the presence of two or more of the following; blood glucose levels higher than 100 mg/dL or diagnosed diabetes; HDL cholesterol <  40 mg/dL in men, <50 mg/dL in women or drug treatment for low HDL-C; blood triglycerides >  150 mg/dL or drug treatment for elevated triglycerides; blood pressure > 130/85 mmHg or drug treatment for hypertension; (2) ages 25 to 70 years

Exclusion criteria were: (1) antibiotic and probiotic treatment in the past month; (2) coexistence of other chronic inflammatory (i.e., chronic hepatitis, asthma, celiac disease, and inflammatory bowel disease) and systemic autoimmune (i.e., systemic lupus erythematosus and rheumatoid arthritis) diseases; (3) steroid therapy in the past 3 months; (4) history of chronic infectious disease (i.e., tuberculosis, human immunodeficiency virus- HIV, hepatitis B virus, and hepatitis C virus); (5) history of COVID-19; (6) pregnancy; and (7) neoplastic disease not in complete remission. Age, sex, and ethnicity matched healthy controls were enrolled based on the same exclusion criteria.

### 4.2. Microbiota and Mycobiota Analysis

Fecal samples were collected while admitted in the hospital or at home following a standardized procedure including antiseptic handling, collection in sterile tubes (without culture media), and immediate freezing at −20 °C. Fecal DNA was extracted using the PureLink Microbiome Purification Kit (Invitrogen, Waltham, MA, USA) according to the manufacturer’s instructions. DNA concentration was determined using a Qubit 4 fluorometer (Thermo Scientific, Waltham, MA, USA). For qPCR analysis, DNA samples were diluted in DNAse free water to a concentration of 3 ng/μL. qRT-PCR measured the relative abundance of intestinal microorganisms in stool DNA isolated from MetSyn patients and healthy controls on a ViiA7^©^ Fast Real-Time instrument (Applied Biosystems, Waltham, MA, USA). The samples were amplified using the bacterial or fungal group-specific primers (16S rDNA and 18S rDNA, respectively) at their specific annealing temperatures. The primers used were selected from the literature [55,56,57], and their sequences are listed in Table 2. Each PCR reaction included 2.5 nM of forward and reversed primer, 9 ng of DNA, and 2x SYBR Green Master Mix (Applied Biosystems). Samples without a DNA template served as negative controls. Samples were incubated at 95 °C for 5 min and then amplified through 40 cycles of 95 °C for 10 s, 60 °C for 30 s, and 72 °C for 1 s.

### 4.3. Metabolite Analysis

Sample preparation for metabolome analysis was prepared as previously described [24]. The weight of the fecal content pellet was adjusted to 0.2 g, and it was resuspended in 1 mL of sterile saline solution and incubated for 2 min at room temperature. Next, the sample was manually homogenized by vigorous shaking (for 4 min) in order to produce a slurry. Samples were centrifuged at 4000 rpm for 1 h at 4 °C, and the supernatant was collected and again centrifuged at 16,000 rpm for 30 min at 4 °C. The supernatant was transferred to a new tube and filtered using a minisart-GF filter membrane (Sartorius, Gottingen, Germany) with a 1 mL sterile plastic syringe. The final step consisted of another filtration using a Whatman-25 mmGD/X0 filter (Merk Millipore, Burlington, MA, USA) and a 1 mL sterile plastic syringe. Metabolite levels (butyrate, acetate, propionate, taurine, succinate, and lactate) were quantified using commercial kits following the manufacturer instructions (Abbexa kit—abx258338 for butyrate quantification and Sigma Aldrich kits for the other metabolites—MAK355, MAK184, MAK065, and MAK086). Optical densities were measured on a spectrophotmoter (Flex3 Station, Molecular Devices, San Jose, CA, USA) and converted to μg/g faeces using the equations provided in the metabolite kits.

### 4.4. Statistical Analysis

Our study data are presented as mean ± SEM and were graphed using the GraphPad Prism 9.0 software. Power analysis was initially performed with a set power (1−β) of 0.90 and α of 0.05 for two groups (Control, MetSyn) tested using difference in means and standard deviation as parameters. To increase the power of the study to 0.95 (α −0.05, β 0.05), additional subjects were included from a second hospital. Differences in microbial relative abundance were assessed using a non-parametric Mann–Whitney test. The * *p* < 0.05 was considered as statistically significant. Statistical significance levels were * *p* < 0.05; ** *p* < 0.01; *** *p* < 0.001. Standardized statistical test methods were used to analyze the results of demography and laboratory tests (biochemistry tests and metabolite levels). Continuous variables were expressed as means ± SD. The analysis of differences between groups was performed by a normality test; a *p*-value ≥ 0.05 was considered to be normal and homogeneous, followed by parametric testing (*t*-test); a *p*-value *<* 0.05 was considered to be statistically significant.

## 5. Conclusions

This pilot study of Romanian patients with MetSyn revealed that gut microbiome alterations were correlated with changes in the profile of related metabolites. MetSyn patients were characterized by a microbiome enriched in *Enterobacteriaceae*, *Turicibacter* sp., *Clostridium coccoides, Clostridium leptum, Saccharomyces* sp., and *Aspergillus* sp. and low in beneficial taxa such as butyrate-producing bacteria (*Butyricicoccus* sp. and *Faecalibacterium prausnitizii*) and the probiotic species *Akkermansia muciniphila*. These microbiome changes were correlated with lower butyrate levels and increased succinate and taurine. In addition, we report changes in the mycobiome associated with MetSyn characterized by an enrichment of *Aspergillus* and *Saccharomyces* species. Our data are the first reported on a Romanian population and are confirming the utility of microbiota derived biomarkers in monitoring the occurrence of underlying pathological conditions in MetSyn patients. Further intervention studies aiming to remodeling the gut microbiome through diet or other medical interventions could identify new personalized strategies in the treatment of MetSyn.

## Figures and Tables

**Table 1:** Patients’ characteristics; BMI—body mass index, HbAc—Glycated hemoglobin, TG—Triglycerides, HDL—high-density lipoprotein, LDL—low-density lipoprotein. %—percentage of patients under treatment.

	Healthy (*n* = 30)	MetSyn (*n* = 30)	*p* Value
Sex	22 females, 8 males	25 females, 5 males	-
Age	56 ± 11.25	62 ± 11.39	0.0446
BMI	25.3 ± 1.255	32 ± 5.39	*p* < 0.0001
HbAc	5.4 ± 0.385	6.6 ± 1.472	*p* < 0.0001
TG	88 ± 20.15	124 ± 54.5	0.0012
HDL	66 ±5.75	49 ± 8.43	*p* < 0.0001
LDL	99 ±19.95	115 ± 35.64	0.0361
Total cholesterol	178 ± 29.56	199 ± 49.92	0.0522
Type 2 diabetes	-	22/30 (73.33%)	-
Hypertension (%)	3/30 (10%)	25/30 (83.33%)	-
Insulin (%)	-	17/30 (56.66%)	-
Metformin (%)	-	13/30 (43.33%)	-
Statins (%)	0	23/30 (76.66%)	-

**Table 2:** Primers used within this study.

Taxonomic Target	Sequence
*Actinobacteria*	TGTAGCGGTGGAATGCGC
	AATTAAGCCACATGCTCCGCT
*Tenericutes*	ATGTGTAGCGGTAAAATGCGTAA
	CATACTTGCGTACGTACTACT
*Verrucomicrobia*	TCAGGTCAGTATGGCCCTTAT
	CAGTTTTCAGGATTTCCTCCGCC
*Bacteroides*	CCTACGATGGATAGGGGTT
	CACGCTACTTGGCTGGTTCAG
*Betaproteobacteria*	AACGCGAAAAACCTTACCTACC
	TGCCCTTTCGTAGCAACTAGTG
*Butyricicoccus* sp.	ACCTGAAGAATAAGCTCC
	GATAACGCTTGCTCCCTACGT
*Gamma proteobacteria*	GCTAACGCATTAAGTACCCCG
	GCCATGCAGCACCTGTCT
*Akkermansia muciniphila*	GCGTAGGCTGTTTCGTAAGTCGTGTGTGAAAG
	GAGTGTTCCCGATATCTACGCATTTCA
*Eubacteria*	ACTCCTACGGGAGGCAGCAGT
	ATTACCGCGGCTGCTGGC
*Lactobacillus*	ACGAGTAGGGAAATCTTCCA
	CACCGCTACACATGGAG
BPP	GGTGTCGGCTTAAGTGCCAT
	CGGACGTAAGGGCCGTGC
*Clostridium leptum*	GCACAAGCAGTGGAGT
	CTTCCTCCGTTTTGTCAA
*Clostridium cocoides*	GACGCCGCGTGAAGGA
	AGCCCCAGCCTTTCACATC
*Ruminococcus* sp.	ACTGAGAGGTTGAACGGCCA
	CCTTTACACCCAGTAATTCCGGA
*Turicibacter* sp.	CAGACGGGGACAACGATTGGA
	TACGCATCGTCGCCTTGGGTA
*Firmicutes*	GGAGCATGTGGTTTAATTCGAAGCA
	AGCTGACGACAACCATGCAC
*Bacteroidetes*	GGAACATGTGGTTTAATTCGATGAT
	AGCTGACGACAACCATGCAG
*F. prausnitzii*	CCCTTCAGTGCCGCAGT
	GTCGCAGGATGTCAAGAC
ARNr 18S	ATTGGAGGGCAAGTCTGGTG
	CCGATCCCTAGTCGGCATAG
*Saccharomyces* sp.	AGGAGTGCGGTTCTTTG
	TACTTACCGAGGCAAGCTACA
*Candida* sp.	TTTATCAACTTGTCACACCAGA
	ATCCCGCCTTACCACTACCG
*Aspergillus* sp.	GTGGAGTGATTTGTCTGCTTAATTG
	TCTAAGGGCATCACAGACCTGTT

## Data Availability

The data presented in this study are available on request from the corresponding author. The data are not publicly available due to privacy/ethical restrictions.

## Supplementary Materials

The following are available online at www.mdpi.com/article/10.3390/metabo12030218/s1, Figure S1: Firmicutes/Bacteroidetes ratio in Met Syn patients (*n* = 30) compared to controls (*n* = 30).
