# Supplementary figures and images for: Microbiome, Mycobiome and Related Metabolites Alterations in Patients with Metabolic Syndrome—A Pilot Study
Source: Metabolites. 2022 Feb 28;12(3):218. doi: 10.3390/metabo12030218 (PMC8951583; doi:10.3390/metabo12030218)

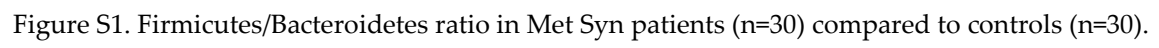

Figure S1. Firmicutes/Bacteroidetes ratio in Met Syn patients (n=30) compared to controls (n=30).

Supplement: Supplementary file 1 [file metabolites-12-00218-s001.zip › metabolites-1546555-supplementary.pdf]
